# Effects of Cr^3+^ Doping on Spinel LiMn_2_O_4_ Morphology and Electrochemical Performance

**DOI:** 10.3390/ijms252413270

**Published:** 2024-12-10

**Authors:** Zhengqing Pei, Jiawei Wang, Haifeng Wang, Kexin Zheng, Qian Wang, Xinjie Zhou, Dehua Ma, Ju Lu

**Affiliations:** 1 School of Materials and Metallurgy, Guizhou University, Guiyang 550025, China; pzq13873253359@163.com (Z.P.);; 2 Guizhou Provincial Key Laboratory of Metallurgical Engineering and Energy Saving, Guiyang 550025, China

**Keywords:** Cr doping, crystal plane orientation, LiMn_2_O_4_

## Abstract

LiMn_2_O_4_, a significant cathode material for lithium-ion batteries, has garnered considerable attention due to its low cost and environmental friendliness. However, its widespread application is constrained by its rapid capacity degradation and short cycle life at elevated temperatures. To enhance the electrochemical performance of LiMn_2_O_4_, we employed a liquid-phase co-precipitation and calcination method to incorporate Cr^3+^ into the LiMn_2_O_4_ cathode material, successfully synthesizing a series of LiCr_x_Mn_2−x_O_4_ (x = 0~0.06). The prepared Cr-doped samples exhibited an excellent spinel structure and a unique truncated octahedral morphology. Additionally, the substitution of Mn^3+^ in LiMn_2_O_4_ by Cr^3+^, coupled with the significantly higher Cr-O bond energy compared to Mn-O bond energy, enhanced the stability of the crystal structure and inhibited the Jahn–Teller effect. Experimental results demonstrated that the optimized LiCr_0.04_Mn_1.96_O_4_ displayed superior electrochemical performance, with a capacity retention rate of 93.24% after 500 cycles under a 0.5C current density; even at 50 °C, the capacity retention rate remained at 86.46% after 350 cycles under a 0.5C current density. The polyhedral morphology formed by Cr doping in LiMn_2_O_4_ offers an effective approach to achieving high-performance LiMn_2_O_4_ cathode materials.

## 1. Introduction

With the sustained global demand for renewable energy and electric vehicles, the development of high-performance, low-cost cathode materials for lithium-ion batteries has become a significant subject in both scientific research and industrial applications [1,2,3,4]. Presently, the primary cathode materials for lithium-ion batteries include spinel-structured LiMn_2_O_4_, olivine-structured LiFePO_4_, and layered LiCoO_2_. Among these, lithium manganese oxide (LiMn_2_O_4_) stands out due to its high theoretical capacity, low cost, and environmental friendliness, making it a highly promising candidate for cathode materials [5,6,7].

Despite its numerous advantages, LiMn_2_O_4_ encounters several critical challenges in practical applications. Notably, under elevated temperatures, LiMn_2_O_4_ exhibits rapid capacity degradation and a short cycle life, significantly limiting its widespread adoption in commercial batteries. Research indicates that the primary cause of this capacity decay is the Jahn–Teller effect induced by Mn^3+^ ions during charge–discharge cycles. This effect leads to lattice distortion, compromising the structural stability of LiMn_2_O_4_ and causing it to progressively lose its electrochemical performance over cycles [8,9,10]. Additionally, the disproportionation reaction of Mn^3+^ during battery cycling generates Mn^2+^, which possesses unstable chemical properties and is susceptible to corrosion by HF in the electrolyte. This results in changes to the elemental composition of the cathode material, further exacerbating its degradation and capacity decay [11,12].

Addressing these challenges is essential for the commercial use of LiMn_2_O_4_. Researchers have implemented diverse modification strategies to enhance LiMn_2_O_4_ materials, primarily focusing on particle morphology control, surface coating, and element doping. Morphology control involves optimizing crystal particle size and orientation by adjusting growth conditions during material synthesis. For instance, Hou et al. [13] designed monocrystalline LiMn_2_O_4_ (TO-LMO) with predominantly exposed (111) faces and minorly truncated (100) and (110) faces. The TO-LMO prepared through a lithium-deficient intermediate phase significantly reduced the disproportionation of Mn^3+^ ions, enhancing structural stability. Using a carbon template sol–gel method, He et al. [14] synthesized LiMn_2_O_4_ with a regular octahedral structure, which, with the abundant presence of a (111) surface, inhibited Mn dissolution, improving cycle stability. The truncated (100) faces facilitated Li^+^ diffusion, enhancing discharge capacity and rate capability. Surface coating typically involves encapsulating the material surface with a stable structure to prevent direct contact between the spinel LiMn_2_O_4_ and the electrolyte, thereby protecting the active material from HF corrosion. Common coating materials include TiO_2_ [15], Al_2_O_3_ [16], ZnO [17], CeO_2_ [18], CeF_4_ [19], SnO_2_ [20], and Nb_2_O_5_ [21].

It is possible to improve the electrochemical performance of LiMn_2_O_4_, particularly via metal cation doping, which is recognized as an effective method. Common cation dopants include Co^2+^ [22], Fe^3+^ [23], Al^3+^ [24], Ni^3+^ [25], Zr^4+^ [26], Mg^2+^ [27], and Ce^4+^ [28]. Xu et al. [22] found that doping Co atoms in LiMn_2_O_4_ provided shorter paths for Li^+^ ion insertion and extraction, accelerating Li^+^ diffusion kinetics. The optimized LiCo_0.05_Mn_1.95_O_4_ exhibited a capacity retention rate of 83.81% after 1000 cycles at 10C. Tao et al. [29] proposed a co-doping strategy with Al and Ni, yielding LiAl_0.1_Ni_0.03_Mn_1.87_O_4_ with well-developed (111), (110), and (100) faces. The co-doping of Al and Ni caused the MnO_6_ octahedra to shrink and the LiO_4_ tetrahedra to expand, significantly enhancing long-term cycle stability. Furthermore, Tian et al. [30] utilized a precipitation–calcination method to dope Cr into lithium manganese spinel oxide, effectively reducing the Mn^3+^ content and stabilizing the spinel framework in an aqueous solution through electrochemical lithium extraction in an HCDI (Hybrid Capacitive Deionization) system. This approach significantly improved the cycling performance, with the prepared LMO-Cr-2 sample maintaining 89.90% of its initial capacity after 500 cycles.

Inspired by the above findings, we adopted a dual strategy of element doping and morphology control to enhance the electrochemical performance of LiMn_2_O_4_. Cr^3+^ was doped during the preparation of the liquid-phase precursor Mn_3_O_4_, and the final synthesis of LiCr_x_Mn_2−x_O_4_ was achieved through calcination with Li_2_CO_3_. We thoroughly investigated the effects of Cr^3+^ doping on the crystal structure, surface morphology, Mn^3+^ content, and electrochemical performance of LiCr_x_Mn_2−x_O_4_. Our findings revealed that the obtained LiCr_0.04_Mn_1.96_O_4_ possesses a unique morphology with both (111) and (100) faces, and Cr doping reduced the Mn^3+^ content. A series of electrochemical tests demonstrated that LiCr_0.04_Mn_1.96_O_4_ exhibited excellent Li^+^ diffusion coefficients, rate performance, and long-term cycle performance.

## 2. Results and Discussion

**Figure 1:**
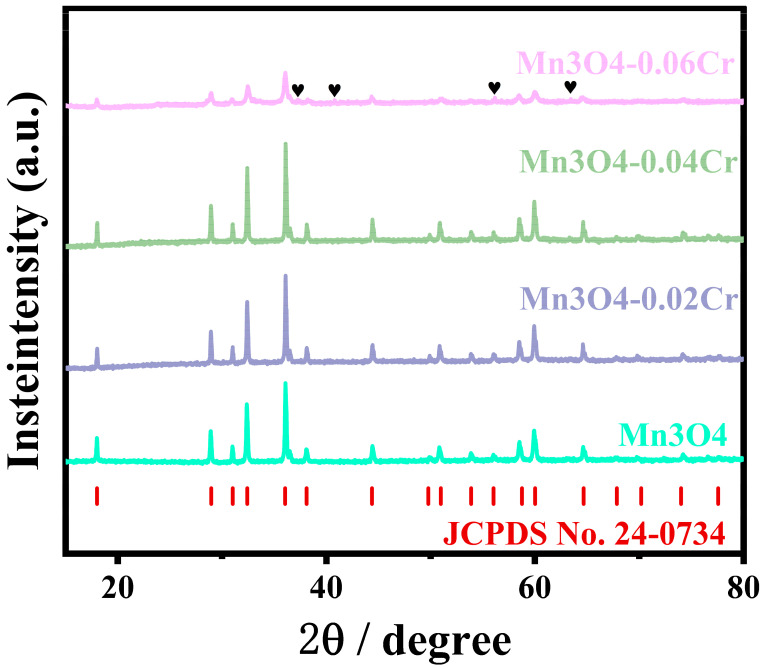
XRD patterns of Mn_3_O_4_-Cr.

Figure 1 shows the XRD patterns of Mn_3_O_4_ synthesized under different Cr doping conditions. As indicated by the figure, the diffraction peaks of all four samples correspond well with those of Mn_3_O_4_, indicating that the main phase of the four precursor samples is Mn_3_O_4_. The samples Mn_3_O_4_, Mn_3_O_4_-0.02Cr, and Mn_3_O_4_-0.04Cr exhibit sharp diffraction peaks and narrow half-peak widths, signifying high crystallinity in these three samples. In contrast, the sample Mn_3_O_4_-0.06Cr has relatively lower diffraction peaks compared to other samples, indicating lower crystallinity. Additionally, there are minor impurity peaks (marked with ♥) present in Mn_3_O_4_-0.06Cr, which are attributed to chromium oxides. These findings suggest that during the preparation of Mn_3_O_4_-0.06Cr, excessive Cr addition led to some Cr not being properly doped into Mn_3_O_4_, resulting in the precipitation of Cr oxides and affecting the crystallization nucleation of Mn_3_O_4_.

**Figure 2:**
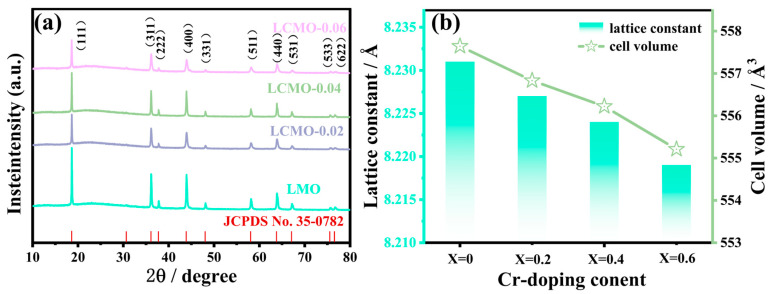
(**a**) XRD patterns of the synthesized samples; (**b**) trend plots of lattice parameters and unit cell volume.

XRD analysis, a rapid and non-destructive technique, was employed to characterize the four samples. As illustrated in Figure 2a, the XRD patterns of all samples corresponded well with the standard card for lithium manganese oxide, exhibiting no significant impurity peaks. This correspondence indicates that the primary component is LiMn_2_O_4_ with an Fd-3m cubic spinel structure, underscoring that the substitution of Cr^3+^ for Mn sites does not alter the spinel structure of LiMn_2_O_4_ [10,31]. A closer examination reveals that the peaks of the LMO sample were sharper and more intense compared to those of the doped samples, suggesting a decrease in crystallinity with Cr^3+^ doping. Among the doped samples, LCMO-0.04 exhibited the strongest peak intensity, indicating superior crystallinity. After refining the XRD patterns of the samples, the lattice parameters were determined as 8.231, 8.227, 8.224, and 8.219 Å, respectively, demonstrating a decreasing trend with increasing doping levels. This decrease suggests that the substitution of Cr^3+^ induces some degree of lattice contraction. This phenomenon can be attributed to the reduction in the content of Mn^3+^ with a high ionic radius of 0.072 nm and a corresponding increase in the content of Mn^4+^ with a smaller ionic radius of 0.067 nm, leading to a reduction in the overall lattice parameter [32].

**Figure 3:**
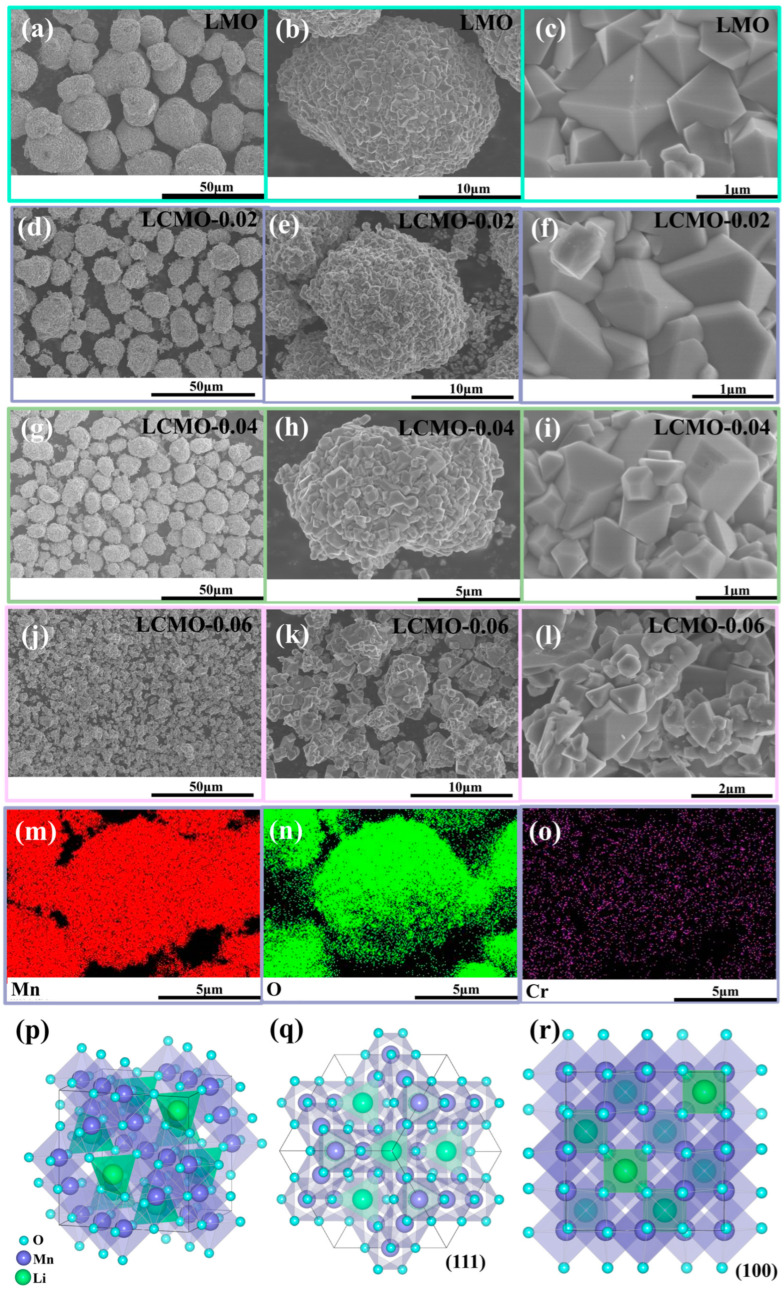
(**a**–**c**), (**d**–**f**), (**g**–**i**), and (**j**–**l**) SEM images of LMO, LCMO-0.02, LCMO-0.04, and LCMO-0.06. (**m**–**o**) EDS images of LCMO-0.04. (**p**–**r**) Schematic diagrams showing the arrangement of LiMn_2_O_4_ with different crystal plane orientations.

Figure 3a, Figure 3d, Figure 3g, and Figure 3j represent the LMO, LCMO-0.02, LCMO-0.04, and LCMO-0.06 samples, respectively. It can be observed that the Cr^3+^ doping significantly impacts the surface morphology of lithium manganese oxide. The LMO sample exhibits particle sizes around 20 μm, while the particle sizes decrease significantly with increasing doping levels. The LCMO-0.04 sample shows a notable reduction in particle size to approximately 10 μm, and the LCMO-0.06 sample exhibits even more dispersed and significantly smaller particles compared to other samples.

A further comparison of Figure 3b,e,h,k reveals that the surfaces of the first three samples are relatively smooth, agglomerated into spherical shapes with good crystallinity, consistent with the previous XRD analysis. In contrast, the LCMO-0.06 sample, influenced by the precursor, shows dispersed and uneven particles. From Figure 3c, it can be observed that the LMO sample, before doping, exhibits a typical perfect cubic spinel structure composed of (111) planes, with relatively complete crystal crystallinity. After doping (Figure 3f), the edges and corners of the spinel become blurred, and the apex no longer appears sharp. Figure 3i shows that in LCMO-0.04, the apex of the spinel completely disappears, presenting a truncated state with a (100) plane, resulting in an overall structure composed of (111) and (100) planes, forming a truncated octahedral shape.

Figure 3p–r illustrate that the (111) planes in the cubic spinel structure have high-density Mn arrangements, exhibiting the lowest surface energy, which helps reduce Mn dissolution in LiMn_2_O_4_ materials. Consequently, {111} planes typically exhibit more stable electrochemical properties. The (100) planes, on the other hand, contain more Li ions, with plane orientations aligned with Li^+^ diffusion pathways, facilitating Li^+^ extraction and insertion [29,33]. In Figure 3l, the spinel particles exhibit varying sizes, with numerous small, incompletely crystallized particles, possibly due to the chromium oxide impurities present in the precursor impeding crystallization during the synthesis of lithium manganese oxide, leading to poor crystal development. Therefore, in subsequent studies, we focus on the LMO and LCMO-0.04 samples to explore the effects of Cr doping on lithium manganese oxide.

Figure 3m–o depict the distribution maps of Mn, O, and Cr elements in LCMO-0.04, clearly showing the uniform distribution of Cr elements in the LiMn_2_O_4_ material through liquid-phase doping.

**Figure 4:**
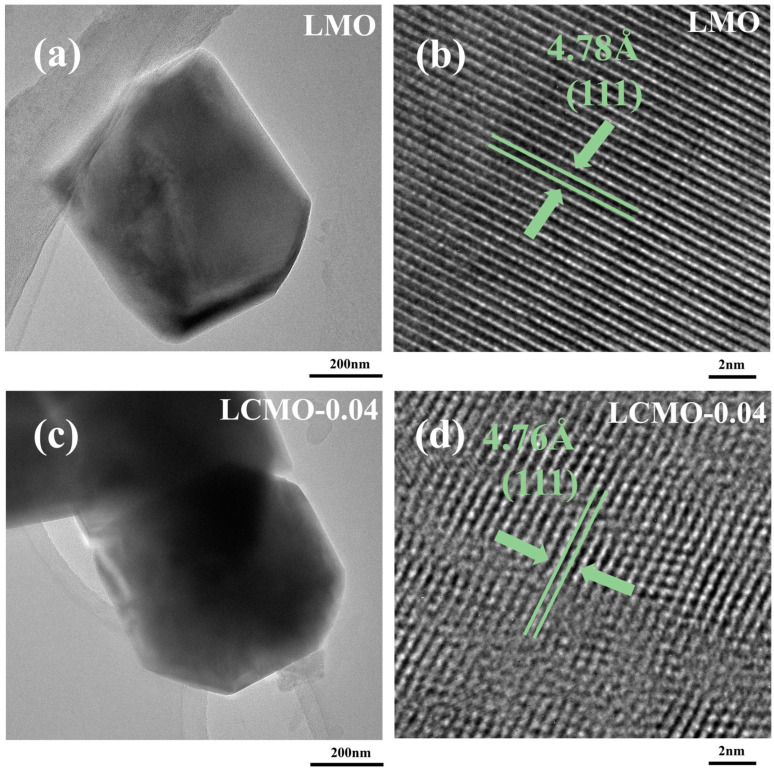
(**a**,**c**) TEM images of LMO and LCMO-0.04. (**b**,**d**) HRTEM images of LMO and LCMO-0.04.

TEM and HRTEM techniques have been employed to provide an in-depth observation of the crystalline structure and surface morphology of the samples. Figure 4a,c show that the single crystal grain sizes of both samples are quite similar, approximately around 800 nm. Notably, the LMO sample displays a spinel-type morphology, whereas the LCMO-0.04 sample exhibits a truncated octahedral morphology.

Comparing Figure 4b and Figure 4d, the interplanar spacings observed in the two samples are 4.78 Å and 4.76 Å, respectively, both corresponding to the (111) crystal planes. The interplanar spacing in the LMO sample is larger than that in LCMO-0.04. This discrepancy may be attributed to the shrinkage of the lattice spacing caused by the Cr^3+^ doping. Additionally, the LMO sample exhibits clearer lattice fringes, while the lattice fringes in LCMO-0.04 are relatively more blurred, indicating that the crystallinity of LCMO-0.04 is lower than that of LMO. These observations collectively highlight the morphological and structural changes induced by the Cr^3+^ doping, with LCMO-0.04 demonstrating a truncated octahedral morphology and reduced crystallinity, likely due to the altered lattice parameters resulting from Cr^3+^ incorporation.

**Figure 5:**
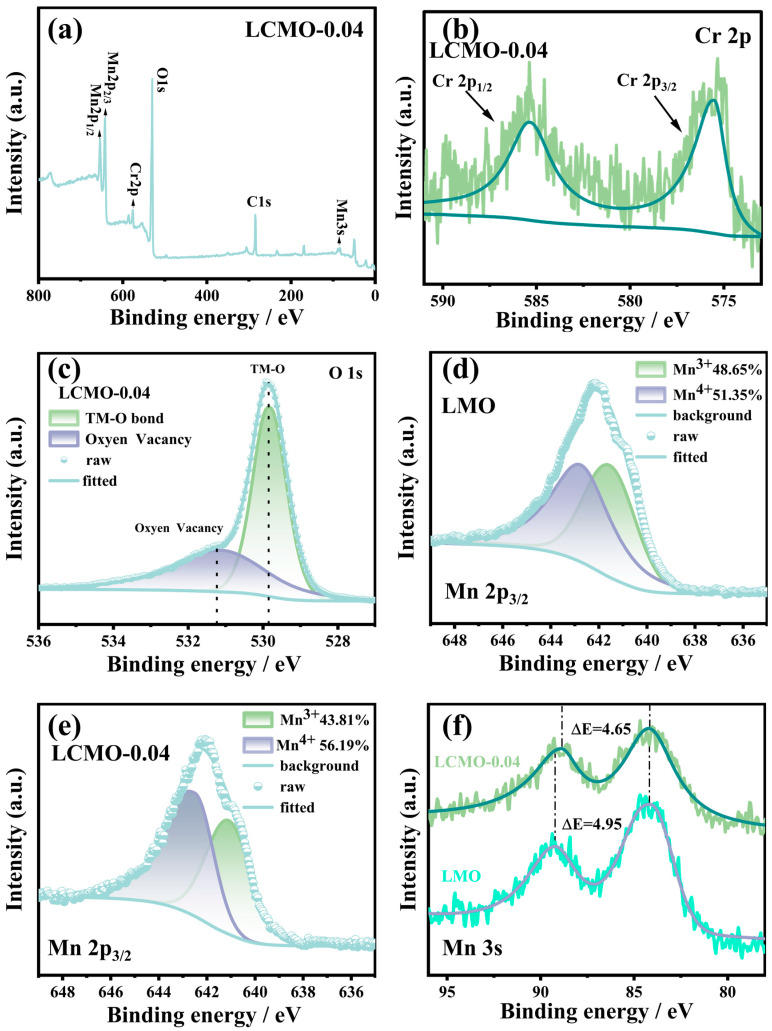
(**a**) XPS full spectrum of the LCMO-0.04 sample. (**b**) Fitted spectra of of Cr 2p inLCMO-0.04 samples. (**c**) Fitted spectra of of O 1s in LCMO-0.04 samples. (**d**,**e**) Fitted fine spectra of Mn 2p3/2 for LMO and LCMO-0.04. (**f**) Fitted spectra of Mn 3s for LMO and LCMO-0.04.

To obtain elemental composition and valence state information on the surface of the samples, we conducted XPS measurements on samples LMO and LCMO-0.04. Figure 5a presents the full spectrum of LCMO-0.04, where diffraction peaks for Mn2p1/2, Mn2p3/2, O1s, and Cr2p are clearly visible. By fitting the fine spectrum of Cr2p for LCMO-0.04, it is observed that the binding energy of Cr2p_3/2_ is approximately around 576.5 eV [34]. This suggests that the majority of Cr elements doped into lithium manganese oxide exist in the +3 valence state, confirming the successful doping of Cr elements into the material [35,36]. Figure 5c shows the fine spectrum fitting of O1s for LCMO-0.04, revealing the presence of not only TM-O (TM=Cr, Mn) but also a small amount of oxygen vacancies in the material. Previous studies have shown that the binding energies of Mn2p_3/2_ for Mn^3+^ and Mn^4+^ typically range from 642.3 to 643.4 eV [37]. By fitting the Mn2p_3/2_ spectra of the LMO and LCMO-0.04 samples, we found their binding energies to be 642.18 eV and 642.23 eV, respectively, both falling within this range. This indicates a mixed valence state of Mn in both samples. Notably, the binding energy of LCMO-0.04 is slightly higher than that of LMO, suggesting a higher average valence state of Mn in LCMO-0.04. The content of Mn^3+^ is closely related to the J-T effect, and controlling the content of Mn^3+^ can help suppress J-T distortion during battery cycling, thereby enhancing structural stability [38,39]. Figure 5d and Figure 5e display the fitting results of the Mn2p_3/2_ orbital, revealing relative contents of Mn^4+^ and Mn^3+^ of 51.35%/48.65% and 56.19%/43.81%, respectively. The doping of Cr^3+^ substitutes Mn^3+^ in LiMn2O4, resulting in a decrease in Mn^3+^ content and an increase in Mn^4+^ content. Studies have shown that a ratio (R, Mn^4+^/Mn^3+^) ≥ 1.18 effectively suppresses the J-T effect [40]. The R values for the two samples were 1.055 and 1.283, respectively, with LCMO-0.04 having an R value greater than 1.18.

Given the multiple splitting binding energies of the Mn2p orbital and the influence of Mn2p_1/2_ on Mn2p_3/2_, relying solely on the Mn2p spectra to determine the chemical state of Mn ions presents certain uncertainties. Therefore, we conducted supplementary tests using the Mn3s orbital [41]. The average oxidation state (AOS) of Mn can be calculated using the formula AOS = 8.956 − 1.126ΔE, yielding AOS values of 3.38 and 3.72 for the two samples, respectively. The increase in AOS after doping is consistent with the results from Mn2p fitting.

**Figure 6:**
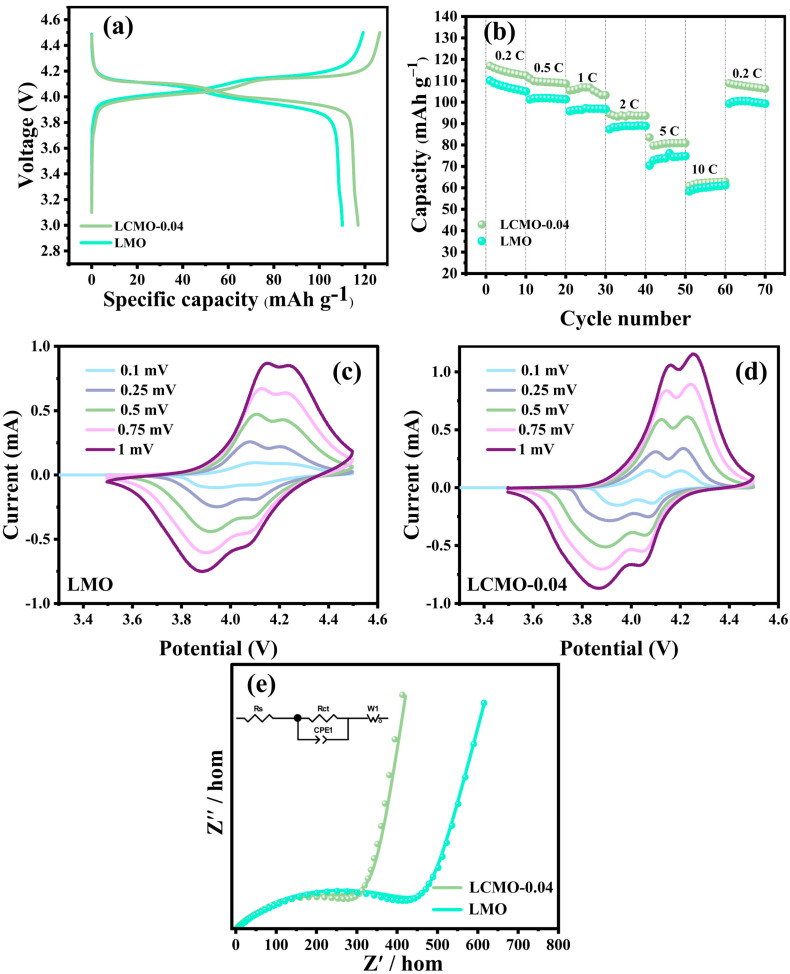
Electrochemical performance plots of the synthesized samples; (**a**) First charge–discharge curves at 0.2 C. (**b**) Rate capability plots. (**c**,**d**) CV curves. (**e**) EIS plots after 5 cycles at 0.5 C.

To comprehensively explore the effect of Cr doping, we conducted a series of electrochemical performance tests on both the LMO and LCMO-0.04 samples within 3.0~4.5 V. The results of these tests are illustrated in Figure 6, with Figure 6a showcasing the initial charge–discharge curves. Notably, both samples exhibit two distinct charge–discharge plateaus located around 3.9 V and 4.1 V, respectively. These plateaus are indicative of the two-step lithium-ion insertion/extraction processes within LiMn_2_O_4_. Specifically, the plateau around 3.9 V correlates with the reaction LiMn_2_O_4_ ⇋ Li_0.5_Mn_2_O_4_ + 0.5Li^+^ + 0.5e^−^, involving the redox process of Mn^3+^/Mn^4+^ and the formation of Li_0.5_Mn_2_O_4_. The second plateau, around 4.1 V, corresponds to the reaction Li_0.5_Mn_2_O_4_ ⇌ 2λ-MnO_2_ + 0.5Li^+^ + 0.5e^−^ [34].

To further assess the performance of the materials under varying current densities, we performed rate capability tests. Figure 6b reveals that as the current density increases from 0.2~10 C, the discharge capacity of both samples exhibits a rapid decline. This phenomenon is attributed to the enhanced polarization of the material with increasing current density, which limits the insertion and extraction of Li^+^, thereby causing capacity decay [42]. Specifically, the average capacities of LMO and LCMO-0.04 during the initial 10 cycles at 0.2 C were 107.14 and 117.02 mAh/g. After the rate capability tests and subsequent return to 0.2 C for another 10 cycles, the average capacities were 100.02 and 108.86 mAh/g, with capacity retention rates of 93.34% and 94.05%, respectively. The slightly higher capacity retention rate of LCMO-0.04 compared to LMO indicates superior electrochemical reversibility for the Cr-doped material.

Figure 6c,d present the CV curves at various scan rates ranging from 0.1 to 1 mV/s. With the increase in scan rate, the voltage gap between the oxidation and reduction peaks gradually widens, indicating an enhanced level of electrochemical polarization. Moreover, the sharpness of the CV peaks reflects a quicker insertion/extraction rate of Li^+^ within the material, as well as enhanced electrochemical activity during charge and discharge [4,32]. By comparing the oxidation and reduction peaks, it is evident that the peaks of the LCMO-0.04 sample are sharper than those of the LMO sample, suggesting better electrochemical reversibility for LCMO-0.04. This phenomenon can be attributed to the smaller particle size of the LCMO-0.04 sample, which provides a larger electrochemical active surface area and thus enables a faster initial electrochemical activation process.

To delve deeper into the electrochemical behavior of the samples, we conducted EIS measurements to analyze the Nyquist plots after five cycles at 0.5 C, as shown in Figure 6e. Table 1 shows the fitting results of the impedance parameters (Rs and Rct). Notably, the Rct values for the LMO and LCMO-0.04 electrodes are 461.8 Ω and 285.8 Ω, respectively. The Rct value for the Cr^3+^-doped LCMO-0.04 sample is significantly lower than that of the LMO sample. Typically, a smaller Rct value is closely associated with superior electrochemical performance [5,43].

**Figure 7:**
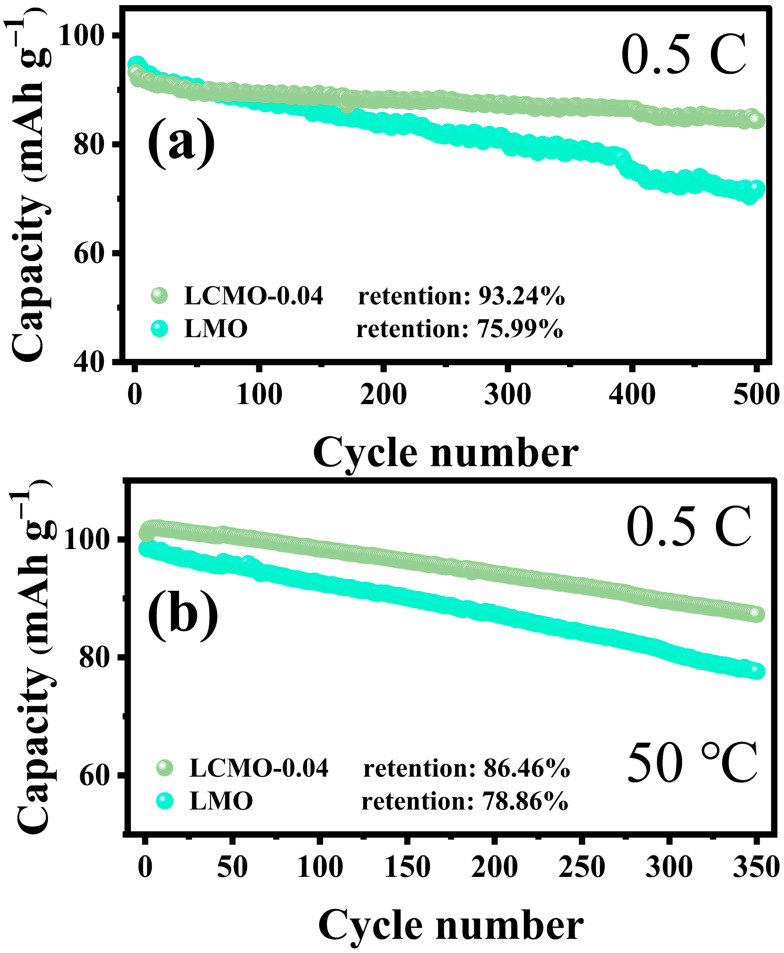
(**a**,**b**) Cycling performance plots.

To assess the impact of Cr^3+^ doping on the long-term cycling performance of LiMn_2_O_4_, we subjected both the LMO and LCMO-0.04 samples to 500 cycles at a rate of 0.5 C at room temperature. The cyclic curves are illustrated in Figure 7a. Initially, the capacities of LMO and LCMO-0.04 were 94.62 and 93.24 mAh/g, respectively, showing a slight difference. However, after 500 cycles, the capacities of LMO and LCMO-0.04 decreased to 71.91 and 84.33 mAh/g, with corresponding cycle retention rates of 75.99% and 93.24%. This indicates that Cr^3+^ doping significantly improves the cycling performance of LiMn_2_O_4_. This enhancement in performance can be primarily attributed to several key factors: Firstly, structural stability is improved. The Cr-O bond energy (1142 kJ·mol^−1^) is stronger than the Mn-O bond energy (946 kJ·mol^−1^). The introduction of Cr-O bonds enhances the overall structural stability of the material, which aids in alleviating structural collapse during prolonged cycling [34]. The robust Cr-O bonds effectively prevent lattice distortion, thereby maintaining structural integrity and reducing irreversible capacity loss. Secondly, the Jahn–Teller effect is inhibited. By substituting Cr^3+^ for some Mn^3+^, the overall content of Mn^3+^ in the material is reduced. The presence of Mn^3+^ can lead to a disproportionation reaction (2Mn^3+^ → Mn^2+^ + Mn^4+^) during cycling. The resulting Mn^2+^ tends to continuously dissolve at the cathode and deposit at the anode, increasing the thickness of the SEI (solid electrolyte interface), enhancing interface impedance, and causing capacity fade, thereby degrading cyclic performance. The doping of Cr^3+^ effectively suppresses this disproportionation reaction, reducing excessive SEI growth and thereby maintaining good electrochemical performance. Lastly, the presence of (100) planes provides more pathways for lithium ion transport, making it easier for Li^+^ to intercalate and de-intercalate [13]. These additional pathways enhance the diffusion rate of lithium ions, thereby improving the rate capability and cyclic stability of the material. 

The cycling performance of spinel lithium manganate at high temperatures has long been a challenge in battery technology, as accelerated decomposition and side reactions in high-temperature environments lead to rapid capacity decay. To address this issue, we conducted long-term cycling tests at 50 °C with a rate of 0.5 C on Cr^3+^-doped samples, with the experimental curves shown in Figure 7b. Initially, the capacities of LMO and LCMO-0.04 were similar, but as the number of cycles increased, a significant difference in capacity decay rate became apparent. After 350 cycles, the capacity retention rate of LCMO-0.04 was 86.46%, while that of LMO was only 78.86%. This indicates that Cr^3+^ doping improves the cycling capability of LiMn_2_O_4_ materials in high-temperature environments.

The GITT provides a direct visualization of the changes in Li^+^ diffusion coefficients during cycling charge and discharge processes. We employed the GITT to assess the LMO and LCMO-0.04 samples, thereby obtaining their respective Li^+^ diffusion coefficients. Initially, the samples were cycled three times between 3 V and 4.5 V at a rate of 0.2 C to activate the battery. After activation, the battery was maintained in an equilibrium state (*E0*) for 1 h. Subsequently, a galvanostatic current pulse was applied for 20 min (*τ*) at 0.2 C, followed by an open-circuit relaxation period of 60 min to attain a steady-state voltage (*Es*). This sequence was repeated until the battery reached the upper voltage limit of 4.5 V, ensuring a comprehensive analysis of the Li^+^ diffusion dynamics for both samples under realistic cycling conditions.

$$D_{{Li}^{+}}=\frac{4}{\Pi \tau }{\left(\frac{m_{B}V_{M}}{M_{B}S}\right)}^{2}\left(\frac{{\Delta E}_{s}}{{\Delta E}_{\tau }}\right))$$

**Figure 8:**
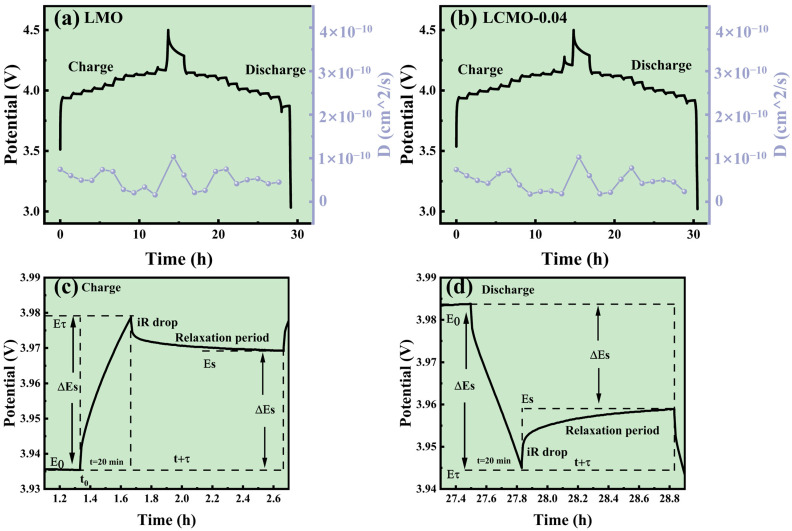
(**a**,**b**) GITT curves and plots showing the changes in lithium ion diffusion coefficients over time for LMO and LCMO-0.04, respectively. (**c**,**d**) The single titration curves for the LMO electrode during charge and discharge.

Δ*Eτ* indicates the voltage change during charge and discharge; Δ*Es* represents the change in equilibrium voltage. Figure 8c,d illustrate the single titration curves for the LMO electrode during charge and discharge, where Δ*Eτ = (Eτ − E0)* and Δ*Es = (Es − E0)*. The charge curve variations are shown in Figure 8a,b, revealing a decrease in *D_Li+_* around 3.9 V and 4.0 V, indicating that phase transitions during charge and discharge lead to slower Li^+^ diffusion [44]. The average *D_Li_^+^* values for LMO and LCMO-0.04 were 4.64× 10^−11^ and 5.05 × 10^−11^ cm^2^/s, respectively, with LCMO-0.04 exhibiting a higher *D_Li_^+^*, signifying superior Li^+^ diffusion rates in the LCMO-0.04 electrode compared to LMO. This observation further validates that doping with Cr^3+^ enhances the electrochemical performance of LiMn_2_O_4_ materials.

## 3. Materials and Methods

### 3.1. Synthesis

**Figure 9:**
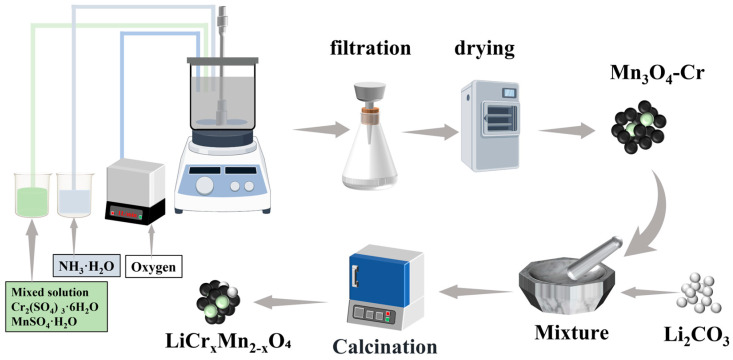
Synthesis of LiCr_x_Mn_2−x_O_4_ samples by figdraw.

A series of LiCr_x_Mn_2−x_O_4_ (x = 0, 0.02, 0.04, 0.06) samples were synthesized via a co-precipitation–calcination method. Initially, the appropriate amounts of MnSO_4_·H_2_O and Cr_2_(SO_4_)_3_·6H_2_O were weighed according to the stoichiometric ratio of Mn to Cr (Mn:Cr = 2−x:x). These compounds were dissolved in deionized water and thoroughly mixed to form a homogeneous solution. In a reaction vessel, deionized water was introduced and stirring commenced. When the temperature reached 50 °C, the Mn-Cr mixed solution, NH_3_·H_2_O, and oxygen were simultaneously pumped into the reactor. The reaction proceeded for 10 h. The resulting precipitates were naturally dried to yield precursor Mn_3_O_4_ containing varying Cr doping levels. Subsequently, the precursors were thoroughly ground and mixed with Li_2_CO_3_. Calcination at 800 °C yielded a series of LiCr_x_Mn_2−x_O_4_, labeled as LMO and LCMO-x (x = 0.02, 0.04, 0.06), respectively. The synthesis flowchart is shown in Figure 9.

### 3.2. Material Characterization

Phase and structural analyses of the samples were conducted using XRD (Bruker D8 Advance, Bruker Corporation in Karlsruhe, Germany) with a Cu target at a scanning rate of 4°/min. The morphology of the samples was observed using SEM (model SU8020, Hitachi High-Tech in Tokyo, Japan) and HRTEM (FEI Tecnai G2 F30, 300 kV, FEI Company in Hillsboro, OR, USA). Surface elemental and valence state compositions were determined by XPS (Thermo Escalab 250Xi, Thermo Fisher Scientific in Waltham, MA, USA).

### 3.3. Electrochemical Testing

The active material sample was mixed uniformly with acetylene black and polyvinylidene fluoride (PVDF) in an 8:1:1 mass ratio. Subsequently, N-methylpyrrolidone (NMP) was added for grinding, which was carried out for a continuous half-hour to obtain a homogeneous mixture. This mixture was then coated onto aluminum foil and dried in a vacuum oven at 120 °C for 12 h to completely remove the NMP. After drying, the mixture was cut into cathode disks with a diameter of 12 mm. Next, using lithium metal as the anode and 1.0M LiPF_6_ in EC:DMC:EMC = 1:1:1 (wt%) as the electrolyte, a button half-cell was assembled. This battery was placed in a CT-4008T-5V battery testing system produced by Shenzhen Neware Electronics Co., Ltd., China, for electrochemical testing. Electrochemical Impedance Spectroscopy (EIS) measurements were conducted using an electrochemical workstation produced by Suzhou DONGHUA Co., Ltd., China. The specific measurement conditions set to collect data within a sine signal of 5 mV and a frequency range from 100 kHz to 0.01 Hz.

## 4. Conclusions

In this work, a series of LiCr_x_Mn_2−x_O_4_ samples were synthesized by incorporating Cr elements at various ratios during the co-precipitation preparation of the precursor Mn_3_O_4_. This study demonstrates that doping with Cr^3+^ significantly impacts lithium manganese oxide, leading to reduced particle size and the formation of polyhedral morphologies with (111) and (100) crystal planes. Appropriate introduction of Cr^3+^ results in a contraction of the unit cell volume, enhancing overall structural stability while reducing the content of Mn^3+^. The optimized LCMO-0.04 sample exhibits excellent Li^+^ diffusion rates and outstanding cyclic performance across both ambient and elevated temperatures. At room temperature, under a 0.5 C rate, it maintains a capacity retention of 93.24% after 500 cycles. Even at 50 °C, after 350 cycles under 0.5 C, the capacity retention remains as high as 86.46%. Therefore, the dual strategy of elemental doping and morphology control effectively suppresses the J-T effect and the dissolution of Mn^2+^, significantly enhancing the electrochemical performance of the material.

## Figures and Tables

**Table 1:** Impedance parameters of LMO and LCMO-0.04.

Samples	LMO	LCMO-0.04
Rs(Ω)	3.158	0.629
Rct(Ω)	461.8	285.8

## Data Availability

Data are contained within the article.

## References

[1] Liu H., Zhu G., Zhang L., Qu Q., Shen M., Zheng H. (2015). Controllable synthesis of spinel lithium nickel manganese oxide cathode material with enhanced electrochemical performances through a modified oxalate co-precipitation method. J. Power Sources.

[2] Fu T., Li Y., Yao Z., Guo T., Liu S., Chen Z., Zheng C., Sun W. (2024). Enhancing Orbital Interaction in Spinel LiNi_0.5_Mn_1.5_O_4_ Cathode for High-Voltage and High-Rate Li-Ion Batteries. Small.

[3] Grimme S., Antony J., Ehrlich S., Krieg H. (2010). A consistent and accurate ab initio parametrization of density functional dispersion correction (DFT-D) for the 94 elements H-Pu. J. Chem. Phys..

[4] Li F., Jiao Y., Yang S., Mao W., Tao Q., Bai C., He E., Li L., Ye T., Li Y. (2024). Electrochemical Activation Inducing Rocksalt-to-Spinel Transformation for Prolonged Service Life of LiMn2O4 Cathodes. Small.

[5] Zhang S., Deng W., Momen R., Yin S., Chen J., Massoudi A., Zou G., Hou H., Deng W., Ji X. (2021). Element substitution of a spinel LiMn_2_O_4_ cathode. J. Mater. Chem. A.

[6] Li H., Xue L., Ni M., Savilov S.V., Aldoshin S.M., Xia H. (2022). Boosting the cycling performance of spinel LiMn_2_O_4_ by in situ MnBO_3_ coating. Electrochem. Commun..

[7] Hou X., Liu X., Wang H., Zhang X., Zhou J., Wang M. (2023). Specific countermeasures to intrinsic capacity decline issues and future direction of LiMn_2_O_4_ cathode. Energy Storage Mater..

[8] Park N.R., Li Y., Yao W., Zhang M., Han B., Mejia C., Sayahpour B., Shimizu R., Bhamwala B., Dang B. (2024). Understanding the Role of Lithium Borate as the Surface Coating on High Voltage Single Crystal LiNi_0.5_Mn_1.5_O_4_. Adv. Funct. Mater..

[9] Tao Y., Liu Q., Guo Y., Xiang M., Liu X., Bai W., Guo J., Chou S. (2022). Regulation of morphology evolution and Mn dissolution for ultra-long cycled spinel LiMn_2_O_4_ cathode materials by B-doping. J. Power Sources.

[10] Li X., Hao S., Wang Z., Zhao C., Wang Z. (2022). Improving the electrical conductivity and electrochemical performance of LiMn_2_O_4_ by Sm gaseous penetration technology. Appl. Surf. Sci..

[11] Deng Y.-F., Zhao S.-X., Xu Y.-H., Nan C.-W. (2015). Effect of temperature of Li_2_O–Al_2_O_3_–TiO_2_–P_2_O_5_ solid-state electrolyte coating process on the performance of LiNi_0.5_Mn_1.5_O_4_ cathode materials. J. Power Sources.

[12] Liu H., Wang J., Zhang X., Zhou D., Qi X., Qiu B., Fang J., Kloepsch R., Schumacher G., Liu Z. (2016). Morphological Evolution of High-Voltage Spinel LiNi_0.5_Mn_1.5_O_4_ Cathode Materials for Lithium-Ion Batteries: The Critical Effects of Surface Orientations and Particle Size. ACS Appl. Mater. Interfaces.

[13] Hou P., Tian Y., Lin Z., Dong M., Li F. (2023). Understanding the key role of {100} exposed crystal facets on the electrochemistry of the spinel LiMn_2_O_4_ cathode. Inorg. Chem. Front..

[14] He J., Zhuang S., Wang Z., Sun G., Pan X., Sun Y., Lu M., Tu F. (2023). Facile preparation of regular truncated octahedral LiMn2O4 cathode with high rate cyclability and stability for Li-ion batteries. J. Alloys Compd..

[15] Ma H., Zeng J., Xie X., Yang P., Ye Y. (2024). Effects of co-coating La_2_O_3_ and TiO_2_ on tissue morphology and electrochemical properties of LiMn_2_O_4_ cathode materials. Ionics.

[16] Pasqualini M., Calcaterra S., Maroni F., Rezvani S.J., Di Cicco A., Alexander S., Rajantie H., Tossici R., Nobili F. (2017). Electrochemical and spectroscopic characterization of an alumina-coated LiMn_2_O_4_ cathode with enhanced interfacial stability. Electrochim. Acta.

[17] Piao J.-Y., Duan S.-Y., Lin X.-J., Tao X.-S., Xu Y.-S., Cao A.-M., Wan L.-J. (2018). Surface Zn doped LiMn_2_O_4_ for an improved high temperature performance. Chem. Commun..

[18] Qureshi Z.A., Tariq H.A., Hafiz H.M., Shakoor R.A., AlQaradawi S., Kahraman R. (2022). Influence of graphene wrapped-cerium oxide coating on spherical LiNi_0.5_Mn_1.5_O_4_ particles as cathode in high-voltage lithium-ion batteries. J. Alloys Compd..

[19] Chen T., Wu H., Zhou D., Zhou Y., Yan W., Song J., Guo J. (2022). The CeF_4_-coated spinel LiNi_0.5_Mn_1.5_O_4_ with improved electrochemical performance for 5 V lithium-ion batteries. J. Mater. Sci. Mater. Electron..

[20] Liang X., Jiang X., Zeng S., Xu W., Lan L., Wu X., Yang D. (2022). SnO_2_ oxide cathode coating: The key ingredient for bi-based polymer quasi-solid-state batteries, enabling stable cycling of high-voltage solid-state LiNi_0.5_Mn_1.5_O_4_/lithium metal battery. J. Electroanal. Chem..

[21] Ji H., Ben L., Wang S., Liu Z., Monteiro R., Ribas R., Yu H., Gao P., Zhu Y., Huang X. (2021). Effects of the Nb_2_O_5_-Modulated Surface on the Electrochemical Properties of Spinel LiMn_2_O_4_ Cathodes. ACS Appl. Energy Mater..

[22] Xu W., Guo S., Li Q., Xia S., Cheng F., Sui F., Qi R., Cao Y., Huang R. (2024). Cobalt doped spinel LiMn2O4 cathode toward high-rate performance lithium-ion batteries. Vacuum.

[23] Lee S.-N., Park D.-H., Kim J.-H., Moon S.-H., Jang J.-S., Kim S.-B., Shin J.-H., Park Y.-Y., Park K.-W. (2022). Enhanced Cycling Performance of Fe-doped LiMn_2_O_4_ Truncated Octahedral Cathodes for Li-Ion Batteries. Chem. Electro. Chem..

[24] Pan B., Zhang H., Weng Y. (2024). Improvement of electrochemical performance of spherical spinel LiMn_2_O_4_ cathode material coated with Al-doped ZnO (AZO) for Li-ion battery. Ionics.

[25] Xu W., Li Q., Sui F., Guo S., Qi R., Yan C., Chen L., Xia S., Guo J., Li Z. (2023). Unveiling the role of Ni doping in the electrochemical performance improvement of the LiMn_2_O_4_ cathodes. Appl. Surf. Sci..

[26] Zhang X.-G., Wu W., Zhou S.-S., Huang F., Xu S.-H., Yin L., Yang W., Li H. (2023). Zr-doping stabilizes spinel LiMn_2_O_4_ as a low cost long cycle life cathode for lithium ion batteries. Chin. Phys. B.

[27] Yu Y., Xiang M., Guo J., Su C., Liu X., Bai H., Bai W., Duan K. (2019). Enhancing high-rate and elevated-temperature properties of Ni-Mg co-doped LiMn_2_O_4_ cathodes for Li-ion batteries. J. Colloid Interf Sci..

[28] Michalska M., Ziółkowska D.A., Jasiński J.B., Lee P.-H., Ławniczak P., Andrzejewski B., Ostrowski A., Bednarski W., Wu S.-H., Lin J.-Y. (2018). Improved electrochemical performance of LiMn_2_O_4_ cathode material by Ce doping. Electrochim. Acta.

[29] Tao Y. (2022). Facile synthesis and electrochemical properties of truncated octahedral Al, Ni dual doped LiMn_2_O_4_ cathode materials. J. Alloys Compd..

[30] Tian G., Gao J., Wang M., Wen X., Liu Y., Xiang J., Zhang L., Cheng P., Zhang J., Tang N. (2024). Structural stabilization of Cr-doped spinel LiMn_2_O_4_ for long-term cyclability towards electrochemical lithium recovery in original brine. Electrochim. Acta.

[31] Cai Y., Huang Y., Wang X., Jia D., Pang W., Guo Z., Du Y., Tang X. (2015). Facile synthesis of LiMn_2_O_4_ octahedral nanoparticles as cathode materials for high capacity lithium ion batteries with long cycle life. J. Power Sources.

[32] Xu W., Zheng Y., Lin L., Lei W., Wang Z., Song H., Cheng Y., Qi R., Peng H., Lin H. (2021). Atomic insights into surface orientations and oxygen vacancies in the LiMn_2_O_4_ cathode for lithium storage. J. Alloys Compd..

[33] Chen Y., Li M., Zhu Q., Bai W., Liu X., Xiang M., Guo J., Liu J. (2024). Enhanced high-rate performance in Zn/Al dual-doped LiMn_2_O_4_ with submicron truncated structure. J. Energy Storage.

[34] Ji Y., Wang N., Guo Y., Guo J., Xiang M., Liu X., Bai W., Bai H. (2023). Preparation of long cycle lifespan spinel LiMn_2_O_4_ cathode material by a dual-modified strategy. J. Alloys Compd..

[35] Biesinger M.C., Payne B.P., Grosvenor A.P., Lau L.W.M., Gerson A.R., Smart R.S.C. (2011). Resolving surface chemical states in XPS analysis of first row transition metals, oxides and hydroxides: Cr, Mn, Fe, Co and Ni. Appl. Surf. Sci..

[36] Li B., Wang M., Zhang Y., Guo Q., Tian R.-N., Chen J., Wang D., Dong C., Mao Z. (2023). Strengthening reversibility at high rate of spinel LiMn_2_O_4_ by aluminum and copper Co-doping for lithium ion battery. Electrochim. Acta.

[37] Liu Q., Zhong L., Guo Y., Xiang M., Su C., Ning P., Guo J. (2021). Facile flameless combustion synthesis of high-performance boron-doped LiMn_2_O_4_ cathode with a truncated octahedra. J. Alloys Compd..

[38] Fu S., Zhang Y., Bian Y., Xu J., Wang L., Liang G. (2023). Effect of Fe^3+^ and/or PO_4_^3–^ Doping on the Electrochemical Performance of LiNi_0.5_Mn_1.5_O_4_ Cathode Material for Li-Ion Batteries. Ind. Eng. Chem. Res..

[39] Liu S., Liu X., Ren D., Li T., Yi L., Liu W., Xu J., Tan T., Zhang J., Hou Y. (2024). Inhibit the strain accumulation for 5V spinel cathode by mitigating the phase separation during high voltage stage. Nano Energy.

[40] Ji Y., Liu H., Guo Y., Guo J., Xiang M., Bai W., Liu X., Bai H. (2022). Improved capacity retention and ultralong cycle performance of Ni-Fe co-doped LiMn_2_O_4_ cathode material at high current densities. Colloids Surf. A Physicochem. Eng. Asp..

[41] Mu J., Wei A., He R., Bai X., Li X., Zhang L., Zhang X., Liu Z., Gao J. (2022). Exploring the synergistic effect of Li+ and Br− co-doping on improving the microstructural and electrochemical performances of LiNi_0.5_Mn_1.5_O_4_ cathode materials. J. Taiwan Inst. Chem. E..

[42] Karunawan J., Suryadi P.N., Mahfudh L., Santosa S.P., Sumboja A., Iskandar F. (2023). Truncated Octahedral Shape of Spinel LiNi_0.5_Mn_1.5_O_4_ via a Solid-State Method for Li-Ion Batteries. Energy Fuels.

[43] Kresse G., Furthmüller J. (1996). Efficiency of ab-initio total energy calculations for metals and semiconductors using a plane-wave basis set. Comp. Mater. Sci..

[44] Velásquez E.A., Silva D.P.B., Falqueto J.B., Mejía-López J., Bocchi N., Del Rio R., Mazo-Zuluaga J., Rocha-Filho R.C., Biaggio S.R. (2018). Understanding the loss of electrochemical activity of nanosized LiMn_2_O_4_ particles: A combined experimental and ab initio DFT study. J. Mater. Chem. A.

